# An unusual presentation of a papillary thyroid carcinoma in the struma ovarii in a 10 year-old girl: A case report

**DOI:** 10.1016/j.ijscr.2018.08.056

**Published:** 2018-09-05

**Authors:** Manouchehr Iranparvar Alamdari, Afshin Habibzadeh, Hossein Pakrouy, Parastoo Chaichi, Sharareh Sheidaei

**Affiliations:** a Department of Internal Medicine, Ardabil University of Medical Sciences, Ardabil, Iran; b Ardabil Nuclear Medicine Center, Ardabil University of Medical Sciences, Ardabil, Iran; c Women’s Reproductive Health Research Center, Tabriz University of Medical Sciences, Tabriz, Iran; d Pathology Department, Ardabil University of Medical Sciences, Ardabil, Iran

**Keywords:** Struma ovarii, Papillary thyroid cancer, Thyroidectomy

## Abstract

**Introduction:**

Struma ovarii is an ovarian tumor with thyroid tissue as its predominant component. It usually occurs in older women and presents with abdominal mass, pain and abnormal bleeding. Most patients are euthyroid, but some reports have noted thyrotoxicosis originating from the malignant struma ovarii.

**Presentation of case:**

A 10 year-old girl presented with tachycardia, normal thyroid exam and thyrotoxicosis. Thyroid scan showed no uptake with increased uptake in right ovary indicative of struma ovarii. The patient underwent oophorectomy and thyroidectomy, Pathology showed papillary thyroid carcinoma limited to the struma ovarii. Following TSH suppressive therapy and treatment with I^131^, she is totally symptom free.

**Discussion:**

Struma ovarii should be a possible diagnosis in any female patient with thyrotoxicosis manifestations with normal thyroid scan and examination.

## Highlights

• Struma ovarii is an ovarian tumor with thyroid tissue as its predominant component.
• Most patients are euthyroid, but some reports have noted thyrotoxicosis originating from the malignant struma ovarii.
• Papillary thyroid carcinoma was reported as the soul mass of struma ovarii in our patient.
• Struma ovarii should be a possible diagnosis in female patients with thyrotoxicosis manifestations with normal thyroid scan and examination.

## 1 Introduction

Struma ovarii, an ovarian tumor, is diagnosed when more than 50% of the teratoma is thyroid tissue [1]. Patients usually refer to gynecologists for abdominal pain, bloating, and abnormal menses and are diagnosed during sonographic evaluations [1,2]. It usually occurs in the older women but it ranges between 22–70 years [3,4].

Struma ovarii’s incidence is less than 2% of mature teratoma [2]. Malignant transformation is less than 5% [1] with the presence of papillary and follicular thyroid carcinoma as the most common type [2]. Most patients are euthyroid and asymptomatic and patients present with symptoms related to the mass [5]. However, thyrotoxicosis have been reported in 5–15% of struma ovarii cases [1,5]. Most malignant struma ovarii have poor iodine uptake with less secreting thyroid hormones [6]. There are some reports of thyrotoxicosis originating from the malignant struma ovarii [4,7].

Here, we report a case of 10 year-old girl with tachycardia, normal thyroid exam and thyrotoxicosis due to the papillary thyroid carcinoma (PTC) limited to the struma ovarii.

The work has been reported in line with the SCARE criteria [8].

## 2 Case presentation

**Fig. 1:**
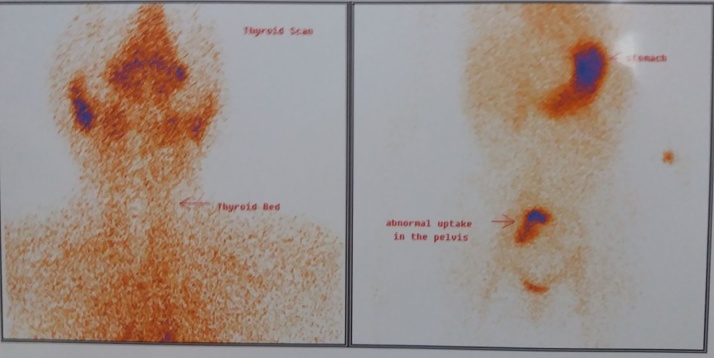
Thyroid scan showing no uptake in the thyroid gland, but increased uptake in the right ovary.

A 10 year-old girl presented with the complaint of palpitation to a cardiologist. She had normal physical examination and laboratory tests, except tachycardia (heart rate = 130 per minute) and low TSH levels (0.005) with normal T3 (9.46) and T4 (145). She was referred to endocrinologist for possible hyperthyroidism evaluation. The thyroid gland was normal size, with no nodularity. She was diagnosed with possible thyrotoxicosis, but due to the normal physical examination, she underwent thyroid scan to rule out possible thyroiditis, which did not show any uptake in the thyroid gland, while there was an increased uptake in the right ovary (Fig. 1). Pelvic trans-abdominal sonography showed a heterogeneous complex solid mass of 113 × 112 × 100 mm with volume of 670 cc in the right ovary with no ascites. The patient had no complaint of abdominal pain or pelvic pain or abnormal uterine bleeding.

**Fig. 2:**
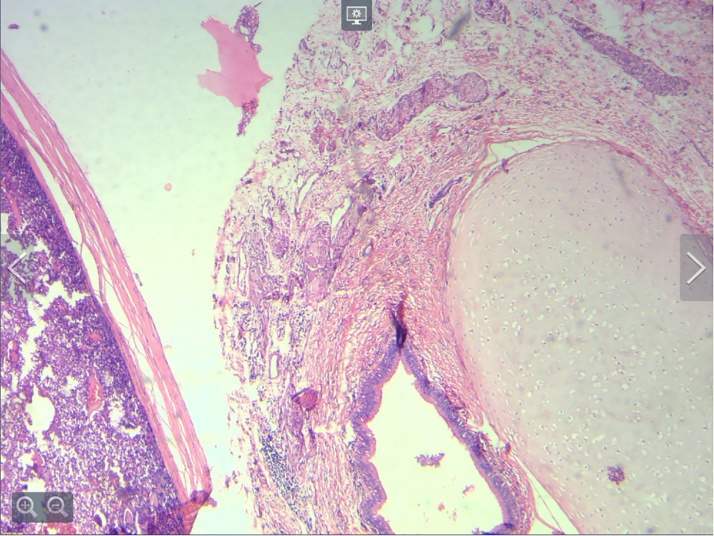
Mature teratoma adjacent to tumor.

She was treated with methimazole 10 mg daily and propranolol 40 mg daily and were candidate for surgery after being euthyroid. The patient was referred to a gynecologist with the possible diagnosis of struma ovarii for further evaluation. She underwent right oophorectomy with the presumption of teratoma combined with thyroid-stimulating hormone (TSH)-suppressive therapy following treatment with I^131^. Total thyroidectomy was performed to permit evaluation for metastatic disease and monitoring for recurrence by thyroglobulin levels. The pathology report of the ovary mass indicated teratocarcinoma with 60% well-differentiated follicular thyroid carcinoma and 40% well differentiated follicular-variant with tumor necrosis, microscopic capsular invasion and peritumoral lymphovascular invasion, considering stage IC of PTC (Fig. 2) and the thyroid gland did not show pathologic features of PTC.

Further evaluation with whole body scan with Iodine 123 (I^123^) showed metastasis to lymph nodes. She had high levels of thyroglobulin and received iodine therapy (150 mCi) twice. In the follow-up whole body scan, there was no trace of iodine uptake and the patient was symptom free.

The patient is now under treatment with levothyroxine 0.1 mg daily. Following 8 months after surgery and iodine therapy, she is totally symptom free.

## 3 Discussion

Struma ovarii can transform into malignant form [1]. It is usually diagnosed in older patients, although it is reported in younger women [3,4] with symptoms of abdominal pain, abdominal mass, ascites and abnormal vaginal bleeding. Hyperthyroidism and thyrotoxicosis have been also reported in 5–15% of the cases [1,5]. Struma ovarii in the young ages are very rare; our case was a 10 year-old girl with clinical and laboratory findings of thyrotoxicosis with no complaint of abdominopelvic origin.

If malignant, the pathology usually show thyroid-related carcinoma with PTC as the most common type. But these patients are usually between 40–50 years old with a mass confined to ovary with median size of 13 cm [9]. Our young patient had PTC limited to the right ovary mass of 11 cm and the thyroid pathology was normal.

It is important to evaluate the thyroid gland to differentiate the mass as primary or secondary tumor due to metastasis. As struma ovarii is diagnosed usually in women at older ages or menopauses, the recommended treatment is hysterectomy and bilateral salpingo-oophorectomy; but unilateral oophorectomy is the choice to preserve fertility in younger patients if there is no extra-ovarian disease. Thyroidectomy is usually recommended to confirm the normal thyroid gland by excluding a primary thyroid carcinoma and potentiate radioablation iodine therapy [10]. Prophylactic thyroidectomy would allow for thyroglobulin monitoring of possible metastases, remained mass or recurrence [10]. However, there are no guidelines in performing prophylactic total thyroidectomy after the diagnosis of thyroid type carcinoma in struma ovarii.

Distant metastatis is very uncommon, while intra-abdominal metastasis can occur in almost 23% of cases [10] including peritoneum, fallopian tubes, contralateral ovary as well as omentum and pelvic and paraaortic lymph nodes [11]. Our patient had regional lymph node metastasis which was eradicated after second iodine therapy.

Patients with malignant struma ovarii have an excellent survival rate. Two large studies by Goffredo et al. [12] and Robboy et al. [9] reported a survival rate of more than 90% for the first ten years and 84.9% at 20 years and 79% and 25 years. Our patient is no disease free for eight months and is followed in routine three months periods.

The pathophysiology of hyperthyroidism in struma ovarii is still unknown. The mechanisms underlying the pathophysiology of functioning struma ovarii is suggested that struma ovarii is an autonomous hormone- secreting tumor or that the ovarian thyroid tissue is stimulated by thyroid-stimulating hormone receptor antibody [13].

## Conflict of interest

We have no conflicts of interest.

## Funding source

No funding source to report.

## Ethical approval

This case report was exempt from ethical approval in our institution.

## Consent

We have parental consent on behalf of the patient for publication of the submitted article and images.

## Author contribution

Manouchehr Iranparvar and Afshin Habibzadeh conceived the idea for the study. All authors were involved in data collection. Hossein Pakrouy performed imaging studies. Sharareh Sheidaei evaluated the pathology findings. Afshin Habibzadeh and Parastoo Chaichi wrote the first draft of the manuscript. All authors edited and approved the final version of the manuscript.

## Registration of research studies

NA.

## Guarantor

Manouchehr Iranparvar and Afshin Habibzadeh.

## Provenance and peer review

Not commissioned, externally peer-review.
